# Some Parasitic Affections of Animals

**Published:** 1892-06

**Authors:** R. R. Dinwiddie

**Affiliations:** Veterinarian, Arkansas Agricultural Experiment Station


### SOME PARASITIC AFFECTIONS OF ANIMALS.

By R. R. Dinwiddie, Veterinarian, Arkansas Agricultural Experiment Station.

Noting the interesting articles from the pen of Dr. Stiles on parasitic affections of animals, I think it may be of interest to record what appear to me to be the most important of these as I find them in this State, from which, perhaps, from the (in places) swampy nature of the soil, and the manner in which all kinds of domesticated animals are allowed to roam at large, parasitism seems to be more common than in the East.

*Distomatoses*—In the Second Annual Report of the Arkansas Agricultural Experiment Station, 1889, I described the extreme frequency of the so-called "Liver Rot" of cattle, in some parts of Arkansas.

I have not been able, as yet, to map out the districts of the State which are infected, but in some four or five counties, at least, practically, all the cattle harbor these liver flukes, whilst in many other counties—as well as in cattle coming from the Indian Territory adjacent—the disease is occasionally met with. As a rule, the number of parasites contained in any one liver is comparatively small—about twenty being the most that I have met with—but my observations have been confined to cattle slaughtered for beef, in which the degree of parasitic invasion is likely to be less than in those in poorer condition. In such beef cattle, even when the liver is badly "rotted," there is no evident signs of ill health, and the carcass usually dresses as well as in other cattle which are not infected. The livers, of course, are always unfit for use; and the "fat caul" is usually darkened, and has to be rejected. The parasites occupy cavities in the substance of the organ, either deeply placed or near the surface, and are surrounded by a thick brownish fluid. Many flukes evidently die in these cavities, which then are found to contain a substance cheesy in consistence and containing the remains of the flukes. In the larger cavities the walls are cartilaginous, or partly calcified.

The regions where the worst infected cattle are raised are flat prairie ranges, subject to overflow on occasions from the St. Francis and White Rivers.

As to the identity of the fluke, I was led, on first finding it, to doubt its identity with the common liver fluke of herbivora (*Dis-*

*toma Hepaticum*), on account of its greater size, as well as from certain seeming differences which it presented from the figures of D. Hepaticum given in those books on Helmenthology to which I then had access.

Still more, from the fact of its apparent non-occurrence in sheep raised in the same localities. It appears that sheep subjected to the same sources of infection remain largely, if not entirely, free from the attacks of this liver fluke. Sheep are not raised to any extent in these localities, but, according to the testimony of the butchers there, those which are raised have generally good livers. I have myself never met with specimens in sheep, but have not had the opportunity to examine a sufficient number to justify a positive assertion as to their being absolutely exempt.

Since my first publication on this subject, fluke disease has been reported in Texas by Prof. Francis (Bulletin No. 18, Texas Agricultural Experiment Station), his fluke doubtless being the same as that which occurs in Arkansas.

Specimens of the latter were sent to Dr. Hassal, who has recently described them under the name of *Fasciola Americana*.

*Nodular disease of the intestines of sheep and cattle.*

This is another rather common affection in sheep raised in this State as well as in Mississippi. In the same report, of which mention has already been made, I described this abnormal condition of the intestine in sheep, surmising correctly, as has since transpired, its cause to be the embryo form of some parasitic worm. The parasite, however, was not found. It has since then been found and described by Dr. Curtice as the embryo of *Oesophagostoma Columbianum*, Curtice. (Animal Parasites of Sheep, 1890).

This modular disease occurs here also in cattle, though more rarely than in sheep. I have also found it in the lungs of both cattle and sheep.

Like the former disease, it does not seem to interfere much with the health of the animal, these modules being found in great numbers in the intestine of very fat and healthy looking sheep. Of course, where use is intended to be made of the intestine, it will necessarily be a source of loss.

*Parasitism in the livers of hogs* is of common occurrence here, and probably throughout the whole State ; as I have been told by butchers in various parts of the State that the livers of hogs are seldom fit for use. In those which I have examined the surface of the liver is dotted over with small white spots, which, on being cut into are found to be small cavities, usually containing a pus-like fluid. There are also tracks leading from these cavities, which would indicate the wandering of the parasite. In some of these little cysts worms are found, in many they are absent; while often there are cicatrices, which indicate their former presence.

This is a round worm, specimens of which on being sent to Dr. Stiles were identified as *Stephanurus Dentatus*, or, "lard worms." I find on consulting the work of Neumann on parasitic diseases, as well as Verrill's lectures on parasites, that these lard or kidney worms have been frequently found in hogs in the United States, but have not found any reference to their common occurrence in the liver. Although the latter authority refers to this parasite as being extremely destructive, I can not find any evidence that it generally acts injuriously on the host, except, in so far as it renders the liver in which it is found unfit for use. As hog's liver is, in most communities, a marketable commodity, this source of loss taken collectively, must be considerable in regions where the disease is so common as it appears to be here.

